# Inducing Biomechanical Heterogeneity in Brain Tumor Modeling by MR Elastography: Effects on Tumor Growth, Vascular Density and Delivery of Therapeutics

**DOI:** 10.3390/cancers14040884

**Published:** 2022-02-10

**Authors:** Constantinos Harkos, Siri Fløgstad Svensson, Kyrre E. Emblem, Triantafyllos Stylianopoulos

**Affiliations:** 1 Cancer Biophysics Laboratory, Department of Mechanical and Manufacturing Engineering, University of Cyprus, Nicosia 1678, Cyprus; charko01@ucy.ac.cy; 2 Division of Radiology and Nuclear Medicine, Department of Diagnostic Physics, Oslo University Hospital, 0372 Oslo, Norway; s.f.svensson@fys.uio.no (S.F.S.); kyrre.eeg.emblem@rr-research.no (K.E.E.); 3 Department of Physics, The Faculty of Mathematics and Natural Sciences, University of Oslo, 0371 Oslo, Norway

**Keywords:** glioblastoma, chemotherapy, perfusion, MRI, MRE, DTI MRI, mathematical modeling, solid stress

## Abstract

**Simple Summary:**

Biomechanical forces aggravate brain tumor progression. In this study, magnetic resonance elastography (MRE) is employed to extract tissue biomechanical properties from five glioblastoma patients and a healthy subject, and data are incorporated in a mathematical model that simulates tumor growth. Mathematical modeling enables further understanding of glioblastoma development and allows patient-specific predictions for tumor vascularity and delivery of drugs. Incorporating MRE data results in a more realistic intratumoral distribution of mechanical stress and anisotropic tumor growth and a better description of subsequent events that are closely related to the development of stresses, including heterogeneity of the tumor vasculature and intrapatient variations in tumor perfusion and delivery of drugs.

**Abstract:**

The purpose of this study is to develop a methodology that incorporates a more accurate assessment of tissue mechanical properties compared to current mathematical modeling by use of biomechanical data from magnetic resonance elastography. The elastography data were derived from five glioblastoma patients and a healthy subject and used in a model that simulates tumor growth, vascular changes due to mechanical stresses and delivery of therapeutic agents. The model investigates the effect of tumor-specific biomechanical properties on tumor anisotropic growth, vascular density heterogeneity and chemotherapy delivery. The results showed that including elastography data provides a more realistic distribution of the mechanical stresses in the tumor and induces anisotropic tumor growth. Solid stress distribution differs among patients, which, in turn, induces a distinct functional vascular density distribution—owing to the compression of tumor vessels—and intratumoral drug distribution for each patient. In conclusion, incorporating elastography data results in a more accurate calculation of intratumoral mechanical stresses and enables a better mathematical description of subsequent events, such as the heterogeneous development of the tumor vasculature and intrapatient variations in tumor perfusion and delivery of drugs.

## 1. Introduction

Glioblastoma multiforme (GBM) is one of the most common primary brain tumors [1,2]. Despite the different treatments developed, it remains a devastating disease with a poor prognosis and an overall survival of 14 to 15 months [3,4]. The response to treatment varies from patient to patient. Thus, the development of patient-specific mathematical models not only enables further understanding of glioblastoma development but also allows the optimization of a patient’s treatment [5].

Mathematical models can be divided into two categories based on the scale at which the tumor is represented. The models can be discrete/stochastic, with an emphasis on the microscopic scale and the interactions at the cellular level, or continuum models, which focus on events taking place at the macroscopic scale [6,7]. Hybrid-multiscale models have also been developed that combine elements of both microscopic and macroscopic models [8]. GBM models most often combine the human brain geometry derived from magnetic resonance imaging (MRI) or computer tomography (CT) with equations accounting for cancer cells’ proliferation and diffusion [9,10,11]. This modeling strategy allows for the prediction of patterns of submicroscopic tumor invasion not detectable by MRI images [9,10,11]. Some models even consider anisotropic diffusion based on data derived from diffusion tensor imaging (DTI MRI), which allows for patient-specific predictions of the shape and evolution of the tumor [12,13,14]. A probabilistic diffusion coefficients scheme in the diffusion reaction equation has also been employed instead of fixed diffusion parameters to improve predictions [15,16]. Furthermore, some studies focus on simulating treatments, such as radiotherapy [17,18,19,20], while others simulate chemotherapy based on the patient’s imaging data [21,22].

The realization that not only biological and brain physiological factors but also biomechanical forces drive brain tumor progression has led to the development of mathematical models that account for tissue biomechanical properties [23,24]. The effect of the biomechanical properties is crucial because tumor progression is associated with the onset and accumulation of mechanical stresses [25,26,27,28]. A source of mechanical stress is solid stress exerted by stromal and cancer cells and the tumor extracellular matrix as a consequence of a growing tumor, which deforms the surrounding tissues [29,30,31,32]. There are also fluid stresses associated with the interstitial and vascular fluid pressure [33,34]. Glioma in silico models consider the effect of stresses with either continuous or discrete approaches [35,36,37,38]. Solid stresses can directly affect glioma cell proliferation and migration [39,40]. They can also induce blood vessel compression and dysfunction, limiting perfusion rates and, thus, oxygen and chemotherapeutic agents’ transport into the tumor [26,41]. Oxygen levels, in turn, affect cancer cell proliferation, tumor growth and invasion as proliferative cells can become invasive under hypoxic conditions [23,35,40,42,43,44,45,46]. Thus, the normalization of brain tumor blood vessels to restore vessels’ hyper-permeability and compression can lead to improved perfusion and therapeutic efficacy [47,48]. The incorporation of tissue mechanics on tumor growth models improves predictions on preclinical models and also helps distinguishing radiation necrosis from tumor progression in patients [49,50].

For a better understanding of the biomechanical tumor microenvironment, a detailed quantification of the mechanical properties of the normal and tumor brain is required. Magnetic resonance elastography (MRE) is a promising imaging technique, which allows for noninvasive quantification of the mechanical properties of tissues by applying external vibrations [51]. Biomechanical properties provide information about tissue stiffness, which is related to the magnitude of mechanical stresses developed in the tumor. Even though MRE has been used for studying brain cancer in patients and animal models [52,53], it has not been used in simulations of brain tumor development, omitting the importance of biomechanical properties in tumor progression.

To this end, we present a mathematical model that incorporates not only conventional anatomical and DTI MRI data but also considers MRE data for a more realistic representation of the biomechanical properties and mechanical stresses in healthy and malignant brain tissues. The model combines the elastography data of a healthy subject’s brain with those of five patients with GBM. Our model simulates tumor progression by assuming that the non-uniform distribution of mechanical stresses promotes proliferation towards low-stress regions [54,55,56,57,58]. This allows for predictions of patient-specific anisotropic tumor growth, non-uniform vessel compression and heterogeneous distribution of functional tumor vessels. Heterogeneous vascular density, in turn, determines chemotherapeutic agents’ transport, posing limits to effective drug delivery [41]. The model highlights the important relationship of elastography data with tumor anisotropic growth, vascular density and chemotherapy delivery and can be a valuable tool for optimizing cancer treatment by using patient-specific noninvasive medical imaging.

## 2. Materials and Methods

### 2.1. Application of MR Imaging Data in the Model

MR imaging was performed on a 3T clinical MRI scanner (Ingenia, Philips Medical Systems, Best, The Netherlands) using a 32-channel head coil. Anatomical T1-weighted, T2-weighted and fluid attenuated inversion recovery (FLAIR) images, as well as DTI MRIs and MRE data, were acquired for five patients, using imaging parameters as in [59], also shown in Table S2. The MRE was performed using a gravitational transducer [60] attached on the side of the head, inducing shear waves of 50 Hz into the brain. The MRE acquisition lasted 5.5 min, with further details about acquisition and processing listed in reference [61]. Patients were between 53 and 75 years (median 60 years), with two female patients and three male patients. All patients had IDH-wildtype glioblastomas, and tumor sizes ranged from 41 cm^3^ to 110 cm^3^ (median 60 cm^3^). Imaging was performed before any treatment. For a healthy subject (a 34-year-old woman), the MRE imaging was extended to cover the entire brain.

Storage and loss modulus values were derived from the MRE data using a localized divergence-free finite element reconstruction [61,62]. The MRE data for both the patients and the healthy subject were converted from a digital imaging and communications in medicine (DICOM) format to Matlab format. Diffusion tensors were derived from the DTI MRI scan of the healthy subject. This was performed using the Diffusion Toolkit (Massachusetts General Hospital, Boston, MA, USA) following a similar procedure as in a previous study [13]. Diffusion tensors were converted to Matlab matrix format too.

A brain geometry employed in a previous study [24] was used here. To reduce computational demands, only the gray matter and white matter regions were included. Generation of the 3D geometry was performed using ScanIP (Simpleware Ltd., Mountain View, CA, USA [24]. The geometry was then imported in COMSOL Multiphysics (COMSOL, Inc., Burlington, MA, USA). Inside the brain geometry, a small spherical tumor, with a radius of 5 mm, acting as the initial tumor seed, was added in the same position for all cases to avoid host tissue mechanical heterogeneities.

A mesh was generated in COMSOL Muliphysics (COMSOL, Inc., Burlington, MA, USA). A finer mesh was used inside and around the tumor domain compared to the rest of the brain in order to improve accuracy and reduce computational cost. The mesh included two types of elements: 1008 prisms that form boundary layers at the tumor boundary and 34,468 tetrahedra for the rest of the geometry.

The storage and loss modulus and diffusion tensors derived from the healthy subject were interpolated in the brain domain. This was done by using a Matlab’s built-in interpolation function (scatteredInterpoland with the method set to natural interpolation) to interpolate the data existing in the Matlab matrixes to the nodes of the finite elements in COMSOL Multiphysics. The same interpolation was used for the patient’s data to the initial tumor seed. This required a deformation of the patient’s data prior to the interpolation, as shown in Figure S1.

For each patient dataset, a rectangular parallelepiped containing the tumor data was extracted. For each patient’s data, the rectangular parallelepiped had the smallest possible dimensions that fitted inside the tumor domain. The parallelepiped was deformed into a cube and then interpolated to the initial tumor seed. For each simulation performed, the tumor seed was subjected to each patient’s elastography data and to the same surrounding elastography data of the normal tissue (derived from the healthy subject). This was done to examine the effect of different tumor elastography properties on the tumor growth.

**Figure 1:**
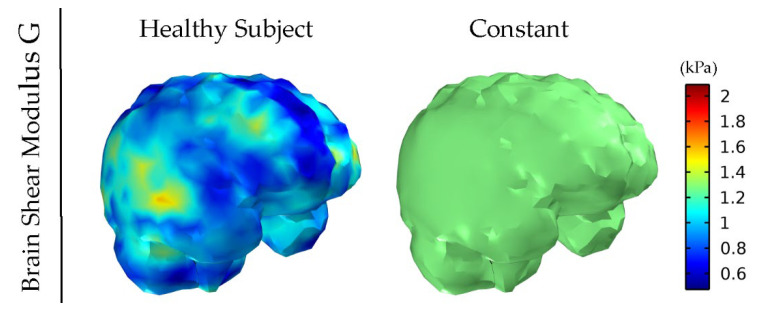
Healthy subject’s shear modulus, G, of the brain derived by MRE. The value for the constant shear modulus case also considered for comparison is the average of the healthy subject’s data.

**Figure 2:**
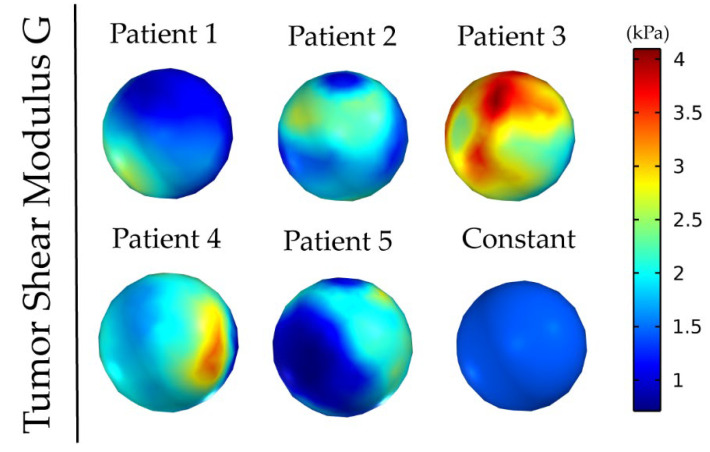
Patients’ tumor shear modulus, G, derived from MRE as it was fitted on the initial tumor seed of the model. The value for the constant shear modulus case is the average value of patient’s 1.

Figure 1 and Figure 2 depict the shear modulus values, G, which are used for the constitutive equation of the normal and brain tumor material model.

The complex shear modulus $G^{*}\left(\omega \right)\;$ can be written as $G^{*}\left(\omega \right){=G}^{\prime }\left(\omega \right)+G\prime \prime \left(\omega \right)$, where $G^{\prime }$ and G″ are the storage and loss modulus calculated by MRE and given by

(1) $$G^{\prime }\left(\omega \right)=G\frac{{{(\omega \tau }_{m})}^{2}}{{1+(\omega \tau }_{m}{)}^{2}},$$

(2) $$G^{\prime \prime }\left(\omega \right)=G\frac{{\omega \tau }_{m}}{{1+(\omega \tau }_{m}{)}^{2}},$$

where ω is the radial frequency, $\tau_{m}$ is the characteristic decay time and G is the shear modulus [63]. In the model, we only considered elastic effects as transient effects due to tissue viscoelastic properties associated with the characteristic decay time were assumed negligible due to the relatively slow growth/deformation rates.

### 2.2. Kinematics of Tumor Growth

Tumor growth is based on principles of continuum mechanics. The deformation gradient tensor, F, was decomposed into two components [64,65].

(3) $${F=F}_{e}\cdot F_{g},$$

where $F_{e}$ is the elastic (reversible) component of F that is related to the stress response of the material. $F_{g}$ is the inelastic (growth, irreversible) component of F. The diagonal components of $F_{g}$ correspond to the growth stretch ratios in the x,y,z direction ($\lambda_{\mathrm{gx}}$,${\;\lambda }_{\mathrm{gy}},\lambda_{\mathrm{gz}})$.

(4) $$F_{g}=\left[\begin{array}{ccc} \lambda_{\mathrm{gx}} & 0 & 0\\ 0 & \lambda_{\mathrm{gy}} & 0\\ 0 & 0 & \lambda_{\mathrm{gz}} \end{array}\right].$$

The elastic component of the deformation gradient tensor is calculated as,

(5) $$F_{e}=F\cdot F_{g}{}^{-1},$$

and the growth stretch ratios are calculated as [54,66],

(6) $$\frac{1}{\lambda_{\mathrm{ga}}}\frac{{d\lambda }_{\mathrm{ga}}}{\mathrm{dt}}{=\Gamma }_{a}r_{g}\;,\;a=x,y,z,$$

where $\lambda_{\mathrm{ga}}$ is the growth stretch ratio in each direction (α = x,y,z) and $r_{g}$ is the mass growth per unit of the current mass. The anisotropic growth multiplier, $\Gamma_{a}$, defines the distribution of the growth term, $r_{g}$, among the three directions (x,y,z) and is written as,

(7) $$\Gamma_{a}{=\Gamma }_{\Sigma }{}^{-1}\exp\left({A\;\sigma }_{\mathrm{aa}}^{s}/k\right),\;a=x,y,z,\;$$

(8) $$\Gamma_{\Sigma }=\exp\left({A\;\sigma }_{\mathrm{xx}}^{s}/k\right)+\exp\left({A\;\sigma }_{\mathrm{yy}}^{s}/k\right)+\exp\left({A\;\sigma }_{\mathrm{zz}}^{s}/k\right).$$

$\sigma^{s}$ is the Cauchy stress, k is the bulk modulus of the tumor and $\Gamma_{\Sigma }$ is defined in a way that $\underset{a}{\sum }\Gamma_{a}=1\;$is satisfied. A is a parameter describing the degree of anisotropy [66]. When A = 0, the Equation (6) becomes

(9) $$\frac{3}{\lambda_{g}}\frac{{d\lambda }_{g}}{\mathrm{dt}}{=r}_{g},$$

the growth stretch ratios, $\lambda_{\mathrm{ga}},\;$become the same and the model accounts for isotropic tumor growth [67]. For A > 0, the larger the value of A, the higher the degree of anisotropy, and growth occurs mostly at the directions of lower stress magnitude [55,56,57,58].

The growth term, $r_{g}$, depends on the oxygen concentration in the tissue, $c_{\mathrm{ox}}$, and the cancer cell density, $T_{\mathrm{cel}}$ [13],

(10) $$r_{g}=\frac{k_{1}{\;c}_{\mathrm{ox}}}{k_{2}{+c}_{\mathrm{ox}}}T_{\mathrm{cel}},$$

where $k_{1}$*,* $k_{2}$ are growth rate parameters.

### 2.3. Stress Balance

According to the biphasic theory for soft tissues [68], the total stress tensor, $\sigma_{\mathrm{tot}}$, can be expressed as the summation of the solid phase stress tensor, $\sigma^{s}$, and the stress tensor, $p_{i}I$, due to the effect of the interstitial fluid pressure $p_{i}$,

(11) $$\nabla \cdot \sigma_{\mathrm{tot}}=0\;\Rightarrow \;\nabla \cdot \left(\sigma^{s}{-p}_{i}I\right)=0.$$

The Cauchy stress tensor, $\sigma^{s}$, is expressed as [69],

(12) $$\sigma^{s}{=J}_{e}^{-1}F_{e}\frac{\partial W}{\partial F_{e}^{T}},$$

where ${J_{e}=\det F}_{e}$ and W is the strain energy density function of the tissue [70].

(13) $$W=\frac{G}{2}\left(I_{1}-3\right)+\frac{k}{2}{\left(J_{e}-1\right)}^{2},$$

where G is the shear modulus calculated from the elastography data and $I_{1}$ is the first invariant of the elastic Green–Cauchy deformation tensor.

### 2.4. Cancer Cell Density

Cancer cell density, $T_{\mathrm{cel}}$**,** was normalized by division with a reference initial value of $10^{7}{\mathrm{cells}/\mathrm{cm}}^{3}$ [71]. Thus, the initial value was set to 1 for the tumor region and to 0 for the host tissue. $T_{\mathrm{cel}}$ is given by the diffusion–reaction equation,

(14) $$\frac{\partial T_{\mathrm{cel}}}{\partial t}+\nabla \cdot {(-D}_{T}\left(x\right)\nabla T_{\mathrm{cel}})=R,$$

(15) $$R_{\mathrm{tumor}}{=r}_{g}=\frac{k_{1}{\;c}_{\mathrm{ox}}}{k_{2}{+c}_{\mathrm{ox}}}T_{\mathrm{cel},}$$

(16) $$R_{\mathrm{host}\;}{=\rho }_{\mathrm{cell}}T_{\mathrm{cel}},$$

where $D_{T}\left(x\right)$ is the inhomogeneous and anisotropic diffusion tensor acquired from the DTI MRI [13,16,72]. In the tumor region, cancer cell proliferation is associated with oxygen supply. The cancer cells that escape the tumor domain due to diffusion were assumed to have a constant proliferation rate, $\rho_{\mathrm{cell}}$.

### 2.5. Interstitial Pressure-Fluid Velocity

Normal and tumor tissues have properties similar to those of a porous medium. According to Darcy’s law, the interstitial fluid velocity is given by

(17) $$v^{f}{=-k}_{\mathrm{th}}\;\nabla p_{i},$$

where k_th_ is the hydraulic conductivity of the interstitial space [73]. The mass balance gives [74,75],

(18) $$\nabla \cdot \left(v^{f}\right){=L}_{P\;}S_{v}\;\left(p_{v}{-p}_{i}\right){-L}_{\mathrm{Pl}\;}S_{\mathrm{vl}}\;\left(p_{i}{-p}_{\mathrm{vl}}\right).$$

The first term of the right-hand side of Equation (18) describes the fluid flux entering from the blood vessels and the second term the flux exiting through the lymphatic system. $L_{P}$ is the blood vessels’ hydraulic conductivity, and $p_{v}$ is the vascular pressure. $L_{\mathrm{Pl}}$, $S_{\mathrm{vl}}$ and $p_{\mathrm{vl}}$ are the corresponding parameters for the lymphatic vessels [76].

### 2.6. Oxygen Transport

The rate of change of oxygen concentration in the tissue was modeled with a convection diffusion equation that includes a source and a sink term [77,78]. The source term is due to oxygen supply from the blood vessels and the sink term describes oxygen consumption by cancer cells:

(19) $$\frac{\partial c_{\mathrm{ox}}}{\partial t}+\nabla \cdot \left(c_{\mathrm{ox}}v^{f}\right){=D}_{\mathrm{ox}}\;\nabla^{2}c_{\mathrm{ox}}-\frac{A_{\mathrm{ox}\;}c_{\mathrm{ox}}}{c_{\mathrm{ox}}{+k}_{\mathrm{ox}}}T_{\mathrm{cel}}{+P}_{\mathrm{erox}}{\;S}_{v}{\;(c}_{\mathrm{iox}}{-c}_{\mathrm{ox}}),$$

where $S_{v}$ is the vascular density, $D_{\mathrm{ox}}$ the oxygen diffusion coefficient, $A_{\mathrm{ox}}$ and $k_{\mathrm{ox}}$ are oxygen uptake parameters, $c_{\mathrm{iox}}$ is the oxygen concentration in the vessels, $v^{f}$ is the fluid velocity and $P_{\mathrm{erox}}$ is the vascular permeability of oxygen defined as the oxygen diffusion coefficient divided by the length of the vessel wall.

### 2.7. Vascular Density

Cancer cell infiltration was studied in our previews work [13]. Thus, in this study, we emphasize the anisotropic tumor growth governed by the effect of elastography data and how that affects stresses and the vasculature. The vascular density was considered as the vascular surface area, S, per unit volume,

(20) $${S=\pi \mathrm{dL}}_{\mathrm{vw}}N,$$

where d and $L_{\mathrm{vw}}$ are the diameter and length of the vessel and N is the number of vessels. By the assumption that the solid stresses do not affect the length or the number of vessels but only the diameter due to compression [41], and by dividing the vascular density with a reference vascular density

(21) $$\frac{S_{v}}{{\;S}_{v0}}=\frac{{\pi \mathrm{dL}}_{\mathrm{vw}}N}{{\pi \mathrm{doL}}_{\mathrm{vw}}N}=\frac{d}{\mathrm{do}}.$$

The functional vascular density can be expressed as [41],

(22) $$S_{v}{=(d/\mathrm{do})\;S}_{v0},$$

where $S_{v0}$ is the vascular density of the host tissue and d/do is the degree of vessel compression assumed to be affected only by the solid stress levels, as described in [41]. The compression is assumed to be affected by the average bulk stress. The average bulk stress is expressed as the trace of the solid Cauchy stress. Initially, the vascular density was assumed to have the value of ${\;S}_{v0}$ in both the tumor and host tissue. In the tumor region, due to the development of stresses, the degree of vessel compression d/do changes as the tumor grows and, thus $S_{v}$ decreases in a stress-dependent manner.

### 2.8. Drug Transport

#### 2.8.1. Drug Transport in the Tumor Interstitial Space

The therapeutic agent can exist in three states: it can travel freely through the interstitial space ($c_{f}$) of the tumor, bind to cancer cells ($c_{b}$) and get internalized by the cells ($c_{\mathrm{int}}$). The equations describing the three states are [79].

(23) $$\frac{\partial c_{f}}{\partial t}+\nabla \cdot \left(c_{f}v^{f}\right){=D}_{f}\nabla^{2}c_{f}{+Q}_{\mathrm{sta}}-\frac{k_{\mathrm{on}}c_{e}c_{f}}{\Phi }{+k}_{\mathrm{off}}c_{b},$$

(24) $$\frac{{\mathrm{dc}}_{b}}{\mathrm{dt}}=\frac{k_{\mathrm{on}}c_{e}c_{f}}{\Phi }{-k}_{\mathrm{off}}c_{b}{-k}_{\mathrm{int}}c_{b},$$

(25) $$\frac{{\mathrm{dc}}_{\mathrm{int}}}{\mathrm{dt}}{=k}_{\mathrm{int}}c_{b}.$$

The free drug that travels in the tumor interstitial space, $c_{f}$, can be transferred due to convection and diffusion, where $D_{f}$ is the diffusion coefficient of the drug in the interstitial space and $v^{f}$ is the fluid velocity. Moreover, the free drug is transferred across the tumor vessel wall ($Q_{\mathrm{sta}}$). The remaining terms describe the binding, unbinding and internalization of the drug; $c_{e}$ is the concentration of cell surface receptors and $k_{\mathrm{on}}{,\;k}_{\mathrm{off}}$ and $k_{\mathrm{int}}$ are the binding, unbinding and internalization rate constants, respectively; Φ is the volume fraction of cells accessible to the drug.

#### 2.8.2. Drug Transport across the Tumor Vessel Wall: Starling’s Approximation

Starling’s approximation was employed for the transport of drugs across the vessel walls

(26) $$Q_{\mathrm{sta}}{=P}_{\mathrm{er}}S_{v}{(C}_{\mathrm{iv}}{-c}_{f}{)+L}_{p}S_{v}{(p}_{v}{-p}_{i}{)(1-\sigma }_{f}{)C}_{\mathrm{iv}},$$

where $P_{\mathrm{er}}$ is the vascular permeability of the drug, $\sigma_{f}$ the reflection coefficient and $C_{\mathrm{iv}}$ is the vascular concentration of the drug expressed as a bolus injection:

(27) $$C_{\mathrm{iv}}{=\exp(–(t–t}_{0}{)/k}_{d}),$$

where $t_{0}$ is the time of drug injection and $k_{d}$ the blood circulation decay. The parameters $L_{p}$, $P_{\mathrm{er}}$ and $\sigma_{f}\;$are expressed as a function of the vessel wall pores and the size of the drug [41,80]:

(28) $$L_{p}=\frac{{\gamma r}_{0}^{2}}{{8\eta L}_{\mathrm{vw}}},$$

(29) $$P_{\mathrm{er}}=\frac{{\gamma \mathrm{HD}}_{0}}{L_{\mathrm{vw}}},$$

(30) $$\sigma_{f}=1–w,$$

where γ is the fraction of the vessel wall surface area occupied by pores, $r_{0}$ the pore radius, η the viscosity of blood plasma and $L_{\mathrm{vw}}$ the thickness of the vessel wall. H and w describe the steric and hydrodynamic interactions of the drug with the pores of the vessel wall that hinder diffusive and convective transport, respectively, and $D_{0}$ is the diffusion coefficient of a particle in free solution given by the Stokes–Einstein equation. By ignoring electrostatic interactions, H and w become [80],

(31) $$H=\frac{6\pi F}{K_{t}},$$

(32) $$w=\frac{{F(2-F)K}_{s}}{{2K}_{t}},$$

where F is the partition coefficient expressed as,

(33) $${F=(1-\lambda )}^{2},$$

where λ is the ratio of the drug size to the vessel wall pore size and $K_{t}$ and $K_{s}$ are expressed as [80]

(34) $$\left(\begin{array}{c} K_{t}\\ K_{s} \end{array}\right)=\frac{9}{4}\pi^{2}\sqrt{2}{\left(1-\lambda \right)}^{-5/2}\left[1+\overset{2}{\underset{n=1}{\sum }}\left(\begin{array}{c} a_{n}\\ b_{n} \end{array}\right){\left(1-\lambda \right)}^{n}\right]+\overset{4}{\underset{n=0}{\sum }}\left(\begin{array}{c} a_{n+3}\\ b_{n+3} \end{array}\right)\lambda^{n}.$$

### 2.9. Solution of Model Equations

At all internal boundaries/interfaces of the computational domains, COMSOL automatically assigned continuity. For the calculation of the displacement fields and stresses, the external surfaces of the brain were considered to have a fixed boundary (u=0). For the transport equations, a no flux boundary condition was assumed at the external surface of the brain. The values of the model parameters are summarized in Table S1 [13,41,45,75,76,78,79,81,82,83,84,85,86,87,88,89].

## 3. Results

### 3.1. Elastography Data Affect Mechanical Stress Distribution and Induce Anisotropic Tumor Growth

**Figure 3:**
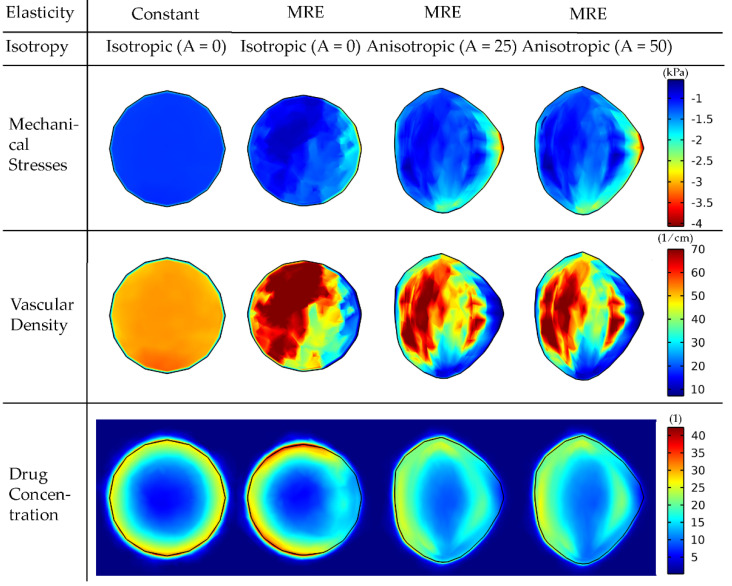
Mechanical stress, vascular density and drug concentration taken up by cancer cells for elastography data of patient 1. A cut plane at the center of the tumor is displayed to visualize the interior of the tumor. Results are presented at day 43 of the simulation. Comparison among isotropic and anisotropic tumor growth by varying the degree of anisotropy (A) is shown. For the constant elasticity case, the average value of the shear modulus was used in the tumor region and the average value of the normal brain for the rest of the brain. A drug of 2 nm in diameter was used to simulate small therapeutic molecules, whereas the tumor vessel wall pore size was set to 200 nm. The bulk mechanical stress is displayed (i.e., the trace of the stress tensor), and the negative sign denotes compression.

We first set out to examine how the incorporation of elastography data by the model affects the magnitude and distribution of intratumoral mechanical stresses and the growth pattern of the tumor. Figure 3 illustrates the comparison of a tumor with a constant averaged shear modulus and a tumor based on the elastography data for isotropic growth, as well as the effect of anisotropic growth.

The incorporation of elastography data into the model results in a non-uniform distribution of mechanical stresses, which, in turn, affects the functional vascular density (due to vessel compression, Equation (22) and, thus, the distribution of the drug taken up by cancer cells. The non-uniform spatial distribution of the vasculature can be observed in Table 1 when comparing the standard deviation of the vasculature of the constant modulus case to that of the cases where MRE data were used. The constant case has a 2.6–2.7 times smaller standard deviation and, thus, a narrower variation in the vascular density values and a more uniform distribution. The standard deviation of the drug’s spatial distribution in the constant modulus case is higher compared to the MRE cases due to the lack of vessel compression at the periphery, where the highest drug transport is observed.

Incorporation of anisotropic growth (i.e., A > 0) allows for the development of more realistic, non-spherical tumor shapes and growth towards the region of lower stresses. Interestingly, an increase in the anisotropic parameter, A, does not have a large effect on the shape of the tumor. The overlap of the tumor shapes is displayed in Figure S2. By evaluating the similarity with the Sorensen–Dice coefficient of the two anisotropic cases, we get a value of 0.9748. Therefore, it seems that the effect of elastography data on the model predictions is dominant compared to the effect of the degree of anisotropy.

**Figure 4:**
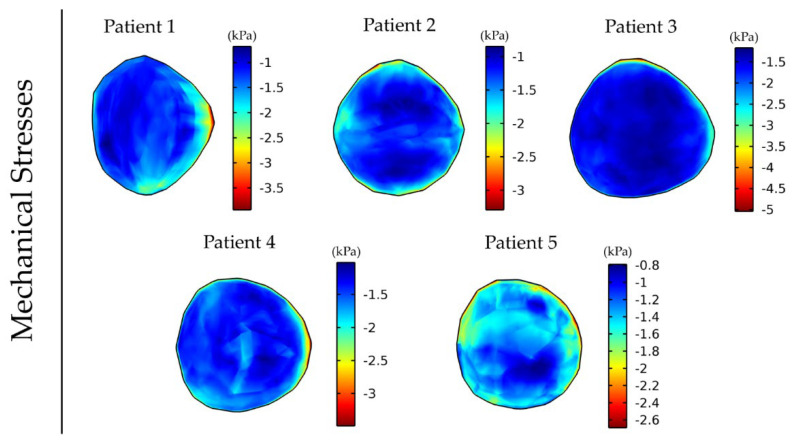
Distribution of intratumor mechanical stress at day 43 of the simulation for anisotropic tumor growth (A = 25). For the tumor region, the corresponding patient’s elastography data were used, and elastography data for a healthy subject were used for the rest of the brain. The bulk stress (trace of the stress tensor) is presented, and the negative sign denotes tissue compression.

Subsequently, we repeated the simulations using the MRE data of the other four patients (Figure 4), with each inducing a different stress distribution in the tumor, which, in turn, caused a different anisotropic tumor growth and, thus, different non-spherical tumor shapes.

### 3.2. Elastography Data Reveal Distinct Functional Vascular Density Distribution among Patients

**Figure 5:**
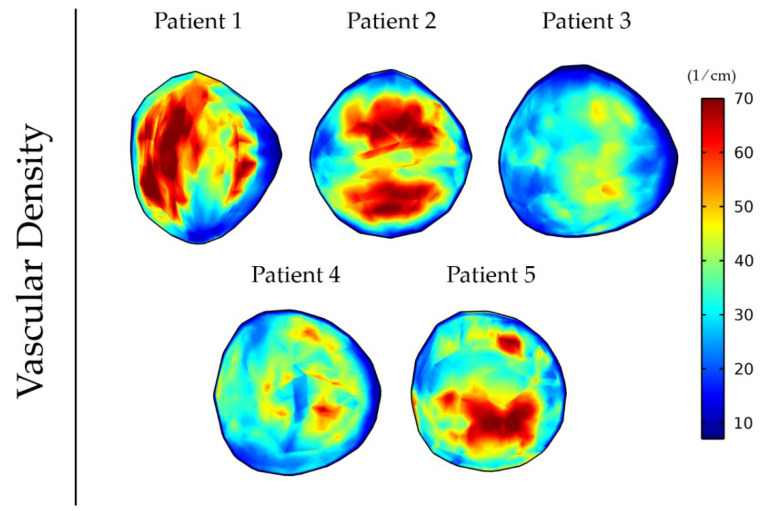
Vascular density at day 43 of the simulation for anisotropic tumor growth (A = 25) when incorporating the elastography data of the five patients.

Vessel compression owing to mechanical stresses causes a reduction in the vessel diameter that limits the area of the lumen available for blood flow. This can have a detrimental effect on tumor perfusion and the functionality of the vessels as the higher the magnitude of stresses the more compressed the vessels become. Figure 5 shows the variation in the magnitude and distribution of the vascular density for the five different patient elastography datasets as a result of the differences in the intratumoral distribution of mechanical stresses (Figure 4). The mean and standard deviation values of the vascular density inside the tumor for the five patients can be found in Table S3.

### 3.3. Elastography Data Affect Intratumoral Drug Distribution

**Figure 6:**
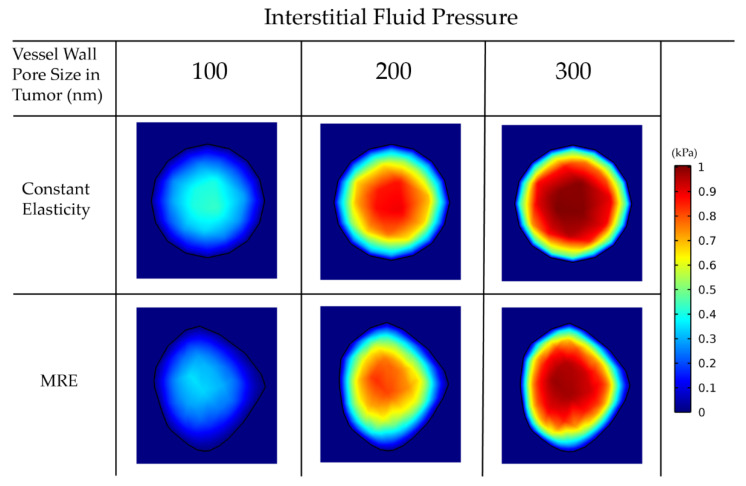
Interstitial fluid pressure for various vessel wall pore sizes. A comparison among the isotropic-constant elastic properties case and the anisotropic-elastography case (for patient 1) is shown.

Abnormal development of vessels during tumor-induced angiogenesis results in vessel hyper-permeability and openings in the tumor vessels wall that can be hundreds of nanometers large [89]. For larger vessel wall pores, the tumor interstitial fluid pressure is uniformly elevated and equals the vascular pressure owing to fluid communication between the vascular and extravascular space of the tumor (Figure 6) [27]. As a result, there is no pressure gradient across the tumor vessel wall nor inside the tumor. Furthermore, there is a steep pressure gradient at the periphery of the tumor as the fluid pressure drops from high values in the tumor interior to normal levels at the interface with the host tissue. For smaller pores in the vessel wall, the distribution of the interstitial fluid pressure is smoother and does not reach the value of the vascular pressure. These observations are well documented in the literature and are typical for the pathophysiology of solid tumors [33,34]. Our data suggest that the incorporation of MRE data does not change the magnitude and elevation of the interstitial fluid pressure.

**Figure 7:**
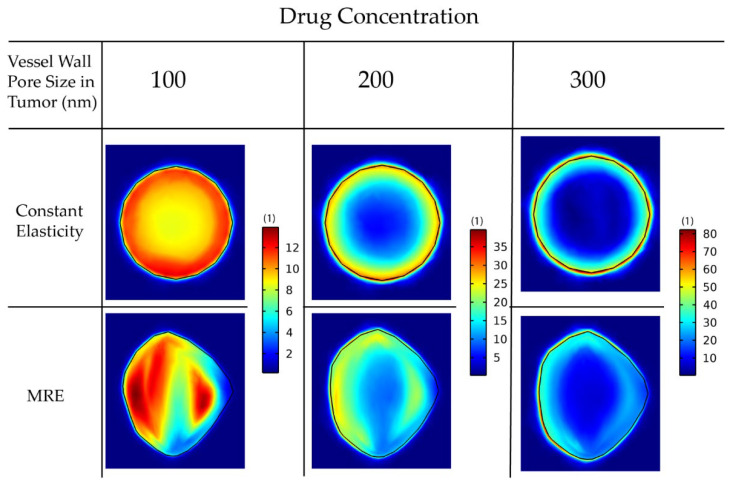
Drug concentration taken up by cancer cells for various wall pore sizes of the tumor vessels. A comparison between the isotropic-constant elastic properties case and the anisotropic-elastography case (patient 1) is shown. All results are displayed at day 43 of the simulation following a drug injection at day 41. The size of the drug is 2 nm.

For larger pores, the lack of pressure gradients eliminates drug transport through convection inside the tumor, and, thus, diffusion becomes the dominant transport mechanism (Equation (26)). Thus, the drug accumulates at the tumor periphery, where both convection and diffusion are prominent, and is washed out from the tumor to the host tissue (Figure 7).

In Table 2, we observe a decrease in the mean values and the standard deviation as we decrease the vessel wall pore size inside the tumor. That means that the establishment of a smooth pressure gradient for smaller vessel wall pore sizes resulted in a more uniform distribution of the drug inside the tumor.

Importantly, incorporation of MRE data can affect model predictions of drug distribution independently of the size of the vessel walls (Figure 7). This is further observed by the mean and standard deviation of the spatial distribution of the drug in the constant versus the MRE cases (Table 2). The use of MRE data in the model leads to predictions of heterogeneous mechanical stress and vascular density distribution. Regions of lower functional vascular density exhibit reduced drug delivery, which results in a heterogenic distribution of the drug.

**Figure 8:**
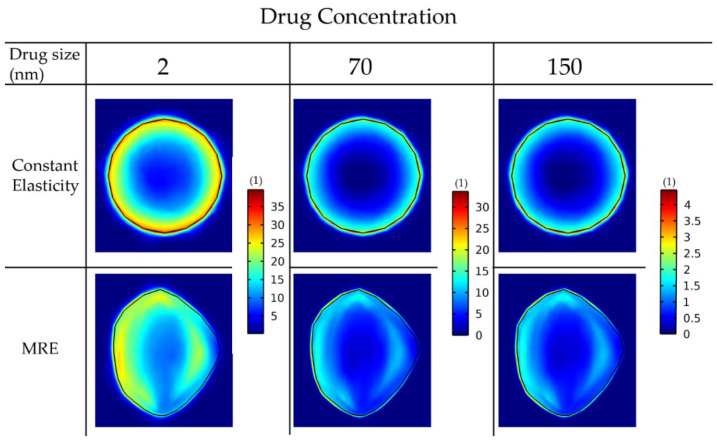
Drug concentration taken up by cancer cells for various drug sizes. A comparison among the isotropic-constant elastic properties case and the anisotropic-elastography case (patient 1) is shown. All results are displayed at day 43 of the simulation following a drug injection at day 41. The vessel wall pore size was set to 200 nm.

Next, we repeated the simulations for the delivery of drugs of different sizes: 2 nm, 70 nm and 150 nm, accounting for small molecules and for nanoparticles (Figure 8).

For the constant elastic properties scenario, the drug distribution is symmetric in the radial direction. This is not the case when the MRE data are included, in which regions of lower functional vascular density exhibit reduced drug delivery. The reduced drug delivery in the MRE cases can be further observed by the decrease in the standard deviation when comparing them with the corresponding constant elasticity values (Table 3). Moreover, smaller drugs can be transferred faster through the pores of the vessels and delivered in larger amounts to cancer cells. Alternative versions of Figure 7 and Figure 8 using the same colorbar for all the drug sizes can be found in Figures S3 and S4.

**Figure 9:**
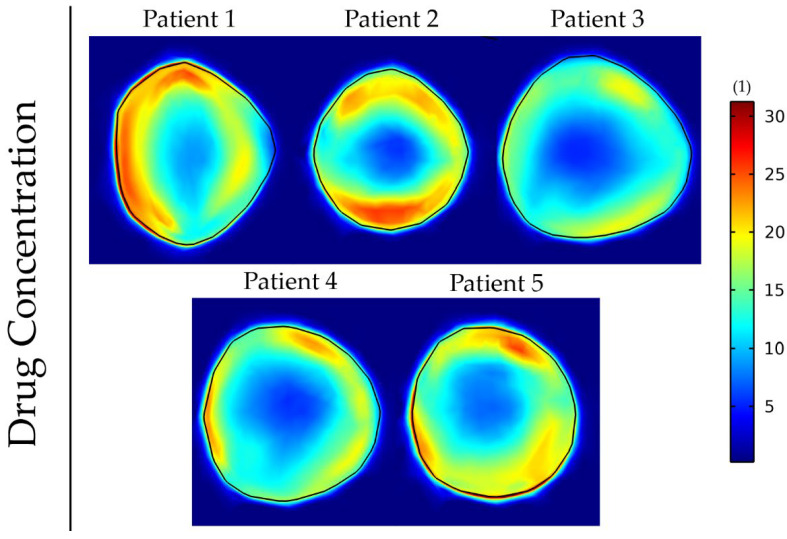
Drug concentration taken up by cancer cells at day 43 of the simulation for anisotropic tumor growth (A = 25). All results are displayed at day 43 of the simulations following a drug administration at day 41. The size of the drug is 2 nm, and the vessel wall pore size was set to 200 nm.

Finally, we employed the elastography data of all the patients to investigate the different patterns of drug delivery within patients (Figure 9).

The results show that the incorporation of patient-specific elastography data can affect the delivery and intratumoral distribution of the drugs. Regions of lower functional vascular density vary among patients, and this results in a distinct drug distribution for each patient. To compare these five cases, we evaluated the fraction of the tumor that receives a drug concentration greater than a specific value (Table 4). This fraction varies by more than 10-fold among the patients. The analysis was also repeated for the cases displayed in Figure 7 and Figure 8 and can be found in Tables S4 and S5, respectively. These results further support that tumor elastic properties can affect drug delivery.

## 4. Discussion

The important role of mechanical forces in tumor progression and therapy is well established [26,27,28,29,30,31,32,41]. Yet, the incorporation of tissue mechanics in mathematical models of brain tumors is not thoroughly studied. Here, we developed a methodology for more accurate calculation of brain tumor mechanics and highlighted its importance for vascular changes and the delivery of therapeutic agents. We included MRE data for a more realistic incorporation of the mechanical properties of both the tumor and host tissue, which led to a more accurate calculation of the intratumoral distribution of mechanical stresses. In addition, to further improve the accuracy of our calculations, we applied a methodology for anisotropic tumor growth, allowing the tumor to grow in non-spherical shapes. We considered that mechanical stresses induce vessel compression and modeled the delivery of drugs of various sizes.

The incorporation of elastography data resulted in a non-uniform distribution of mechanical stresses. The incorporation of anisotropic growth allowed the development of a more realistic non-spherical tumor shape and growth towards the regions of lower stresses. The non-uniform mechanical stresses induced a non-uniform distribution of vascular density due to vessel compression. This resulted in a non-symmetric distribution of drugs where regions of lower functional vascular density exhibited reduced drug delivery. Stress distribution, vascular density distribution and drug delivery are unique for each patient’s MRE data, and, thus, the inclusion of MRE data allows patient-specific predictions.

Smaller pores of the vessel wall induced a smoother distribution of interstitial fluid pressure. The incorporation of MRE data did not change the magnitude and elevation of interstitial fluid pressure. Smoother pressure gradients caused a more uniform distribution of drug inside the tumor. In addition, our results suggest that smaller drugs can be transferred faster through the pores of the vessels and delivered in larger amounts to the cells compared to larger drugs. Overall, our findings can be used to improve treatment response assessment and evaluation of pharmacological strategies as MRE is a noninvasive imaging technique that can be added to patients’ MR examination. MRE is an emerging imaging technique that has been used in several studies of patients with brain tumors [90]. Currently, there is no commercial system available for brain MRE, limiting its potential as a routine part of brain cancer imaging. The patients in our study were all imaged prior to any treatment, but using MRE on patients after surgical tumor resection is clinically feasible and currently ongoing at our institution as part of a clinical trial and with minimal implications (NCT03951142).

Several simplifying assumptions were made in this study. For the host tissue, elastography data of a healthy subject were used because the clinical patient scans did not cover the entire brain, only 4.65 cm, covering the tumor. Because the patients differ from healthy subjects in terms of MRE values [59], more accurate results would have been obtained if the specific patient’s elastography of the host tissue was used. In addition, patients’ MRE values were obtained at one time point during tumor development and, thus, in the model, the elastic properties were assumed constant during tumor progression. That is not usually the case. Due to changes in the cellular and extracellular matrix components, the compression of the tumor and the host tissue changes during tumor progression. These effects can result in changes in the stiffness of the tumor [26,32,91,92]. The incorporation of temporal variations in the elastic properties would be expected to change our results quantitatively. However, the main conclusions of our study concerning the role of mechanical forces in tumor vasculature and drug delivery are not expected to be altered by this. Furthermore, the evolution of the tumor in the model was related to the simulation time-step and not the actual time for tumor growth. This was done due to the absence of individual tumor growth rates. Moreover, the timepoint of the injection of chemotherapy was also an assumption. The same timepoint of injection was used for all the cases to enable direct comparison among different simulations. Finally, the isotopic neo-Hookean constitutive equation might not be sufficient to fully describe the mechanical response of brain tumors because of the heterogenous structure of the GBM. However, studies have shown that the state of stress of the tumor is largely determined by the properties of the host and tumor tissue and not from the selection of the constitutive equation being used [30].

## 5. Conclusions

The presented methodology and results led to the conclusion that incorporating the tissue elastic properties assessed by MRE and anisotropic growth into mathematical models can result in more accurate predictions of the distribution of mechanical stresses in tumors. This produces an improved mathematical description of subsequent events that are closely related to the development of mechanical stresses, including the heterogeneity in the functional vasculature of the tumor and intrapatient variations in tumor perfusion and delivery of drugs.

## Figures and Tables

**Table 1:** Mean and standard deviation values of the spatial distribution of the vascular density and drug concentration in the tumor for the 4 cases considered in Figure 3.

		Constant-Isotropic (A = 0)	MRE-Isotropic (A = 0)	MRE-Anisotropic (A = 25)	MRE-Anisotropic (A = 50)
Vascular Density (1/cm)	Mean	50.263	47.560	46.933	45.524
	Standard Deviation	7.014	18.942	18.267	19.261
Drug Concentration	Mean	18.954	17.726	18.471	18.282
	Standard Deviation	6.292	5.970	4.436	4.723

**Table 2:** Mean and standard deviation values of the spatial distribution of drug concentration in the tumor for the 6 cases considered in Figure 7.

	Vessel Wall Pore Size in Tumor (nm)	100	200	300
Constant Elasticity Drug Concentration	Mean	10.185	18.954	24.223
	Standard Deviation	0.919	6.292	14.162
MRE Drug Concentration	Mean	9.361	18.282	23.736
	Standard Deviation	2.656	4.723	8.886

**Table 3:** Mean and standard deviation values of the drug concentration inside the tumor domain of the 6 cases of Figure 8.

	Drug Size (nm)	2	70	150
Constant Elasticity Drug Concentration	Mean	18.954	9.221	1.201
	Standard Deviation	6.292	4.413	0.575
MRE Drug Concentration	Mean	18.282	8.957	1.185
	Standard Deviation	4.723	3.114	0.413

**Table 4:** Fraction of the tumor that receives a drug concentration greater than 20 (dimensionless units) for the 5 patients at day 43 of the simulation.

	Patient 1	Patient 2	Patient 3	Patient 4	Patient 5
Volume Fraction (Drug Concentration > 20)	0.375	0.173	0.027	0.038	0.206

## Data Availability

All data supporting the findings of this study are available in the paper and the Supplementary Materials.

## Supplementary Materials

The following are available online at https://www.mdpi.com/article/10.3390/cancers14040884/s1, Table S1: Values of model parameters, Table S2: Scan parameters for patients and healthy subject, Table S3. Mean and standard deviation values, vascular density and drug concentration inside the tumor domain of the 5 patients at day 43 of the simulation, Table S4. Fraction of the tumor that receives drug concentration greater than 20 for the 6 cases of Figure 7, Table S5. Fraction of the tumor that receives drug concentration greater than 20 for the 6 cases of Figure 8, Figure S1. Deformation and interpolation of the patient’s data to the initial tumor seed: (A) T1c MRE space used to locate the tumor region of each patient; (B) tumor data of each patient; (C) deforming the data into becoming a cube; (D) interpolating the data of the cube to each initial tumor seed; (E) initial tumor seed with data, Figure S2. Overlap of the tumor shapes displayed in Figure 3 for the isotropic (A = 0) case and anisotropic (A = 25 and A = 50) cases, Figure S3. Second version of Figure 7 where the same colorbar was used for all figures, Figure S4. Second version of Figure 8 where the same colorbar was used for all figures. References [13,41,45,75,76,79,81,82,83,84,85,86,87,88,89] are cited in the Supplementary Materials.
