# Supplementary material for: Inducing Biomechanical Heterogeneity in Brain Tumor Modeling by MR Elastography: Effects on Tumor Growth, Vascular Density and Delivery of Therapeutics
Source: Cancers (Basel). 2022 Feb 10;14(4):884. doi: 10.3390/cancers14040884 (PMC8870149; doi:10.3390/cancers14040884)
Supplement: Supplementary file 1 [file cancers-14-00884-s001.zip › cancers-1596073-supplementary.pdf]

cancers

MDPI

Supplementary Material: Inducing Biomechanical Heterogeneity in Brain Tumor Modeling by MR Elastography: Effects on Tumor Growth, Vascular Density and Delivery of Therapeutics

Constantinos Harkos, Siri Fløgstad Svensson, Kyrre E. Emblem and Triantafyllos Stylianopoulos *

Table S1. Values of model parameters.

Parameter	Description	Value	Reference
k_{th}	hydraulic conductivity of tumor	3.8×10^{-12} m²·Pa⁻¹·s⁻¹	[1]
c_{iox}	oxygen concentration in the vessels	0.2 mol·m⁻³	[2,3]
D_{ox}	oxygen diffusion coefficient	1.55×10^{-4} m²·day⁻¹	[2]
A_{ox}	oxygen uptake parameter	2200 mol·m⁻³·day⁻¹	[2,4]
k_{ox}	oxygen uptake parameter	0.00464 mol·m⁻³	[2,4]
k_1	growth rate parameter	0.032 day⁻¹	[5]
k_2	growth rate parameter	0.0083 mol·m⁻³	[4]
ν_{Tumor}	Poisson's ratio (tumor)	0.25	[6]
ν_{Normal}	Poisson's ratio (host)	0.2	[6]
ω	radial frequency	100π rad·s⁻¹	--
ϱ_{cell}	constant proliferation rate	0.012 day⁻¹	[7]
p_{v_tumor}	vascular pressure in tumor	1 kPa	[8]
p_{v_Normal}	vascular pressure in host tissue	2 kPa	[8]
p_{vl}	lymphatic pressure	0	[6]
S_{v0}	vascular density of host tissue	70 cm⁻¹	[8]
$L_{Pl} S_{vl}$	permeability of lymphatics	0.05 (mmHg·s)⁻¹	[8]
c_e	receptor concentration	0.01 mol·m⁻³	[9,10]
Φ	volume fraction of tumor accessible to drug	0.3	[9,10]
k_{on}	binding rate constant	1.296×10^6 m³·mol⁻¹·day⁻¹	[11,12]
k_{off}	dissociation rate constant	691.2 day⁻¹	[11,12]
k_{int}	internalization rate constant	3.7 day⁻¹	[11,12]
t_0	time of drug injection	41 day	--
$D_{f\_rs=1nm}$	drug diffusion coefficient (1nm)	1×10^{-6} cm²·s⁻¹	[13]
$D_{f\_rs=35nm}$	drug diffusion coefficient (35nm)	0.5×10^{-8}cm²·s⁻¹	[13]
$D_{f\_rs=75nm}$	drug diffusion coefficient (75nm)	0.5×10^{-9} cm²·s⁻¹	[13]
k_d	blood circulation decay	0.417 day⁻¹	[14]
L_{vw}	vessel wall thickness	5×10^{-6} m	[6]
η	plasma viscosity at 310K	1.3×10^{-3} Pa·s	[15]
γ_{tumor}	fraction of vessel wall surface area occupied by pores (tumor)	1×10^{-3}	[14]
γ_{Normal}	fraction of vessel wall surface area occupied by pores (host)	1×10^{-4}	[14]
r_{0_host}	vessel wall pore radius (host)	3.5 nm	[16]
r_{0_tumor}	vessel wall pore radius (tumor)	50–150 nm	[16]

Table S2. Scan parameters for patients and healthy subject.

Sequence	Repetition time (ms)	Echo time (ms)	Acquisition matrix	Resolution (mm^3)	Additional information
T_2-weighted	3000	80	420 × 270 × 28	0.6 × 0.6 × 4	Turbo-spin echo sequence
FLAIR	4800	320	252 × 249 × 183	1 × 1 × 1	Turbo-spin echo sequence, inversion time = 1650 ms
T_1-weighted	5.2	2.3	256 × 256 × 368	1 × 1 × 1	3D inversion recovery turbo field echo, flip angle = 8°, shot interval = 3000 ms, inversion delay = 853 ms
Diffusion Tensor Imaging	9800	60	94 × 94 × 50	2.5 × 2.5 × 2.5	Spin echo, single-shot, echo-planar imaging readout, sensitivity encoding 2, 15 gradient directions, b-values 0 s/mm^2 and 800 s/mm^2
MR Elastography	295	12	72 × 70 × 15	3.1 × 3.1 × 3.1	Motion-encoding gradient strength 13.2 mT/m, sensitivity encoding 2

Table S3. Mean values and standard deviation vascular density and drug concentration inside the tumor domain of the 5 patients at day 43 of the simulation.

		Patient 1	Patient 2	Patient 3	Patient 4	Patient 5
Vascular Density (1/cm)	Mean	46.933	40.110	29.515	32.882	38.723
	Standard Deviation	18.267	16.336	11.619	12.976	18.025
Drug Concentration	Mean	18.471	16.159	13.352	14.291	16.265
	Standard Deviation	4.436	4.080	4.361	3.229	4.932

Table S4. Fraction of the tumor that receives drug concentration greater than 20 for the 6 cases of Figure 7.

	Vessel Wall Pore Size in Tumor (nm)	100	200	300
Volume Fraction (Drug Concentration > 20)	Constant Elasticity	0	0.464	0.560
	MRE	0	0.373	0.666

Table S5. Fraction of the tumor that receives drug concentration greater than 20 for the 6 cases of Figure 8.

	Drug Size (nm)	2	70	150
Volume Fraction (Drug Concentration > 20)	Constant Elasticity	0.464	0.001	0
	MRE	0.373	0.002	0

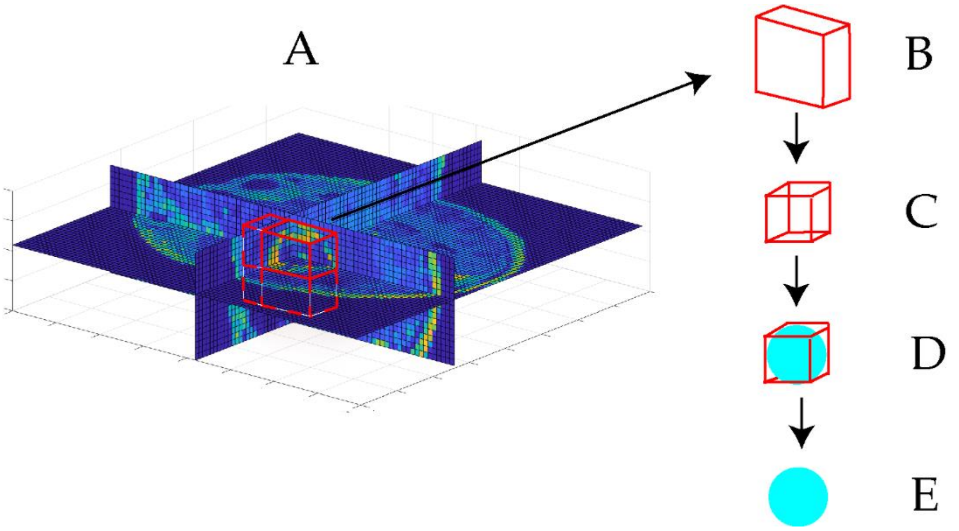

Figure S1. Deformation and interpolation of the patient's data to the initial tumor seed: (**A**) T1c MRE space used to locate the tumor region of each patient; (**B**) tumor data of each patient; (**C**) deforming the data into becoming a cube; (**D**) interpolating the data of the cube to each initial tumor seed; (**E**) initial tumor seed with data.

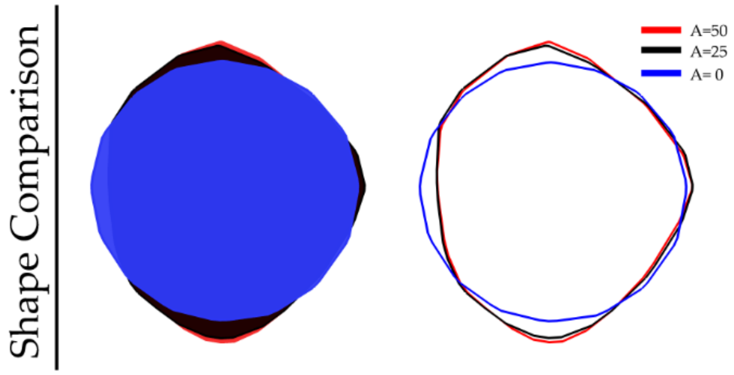

Figure S2. Overlap of the tumor shapes displayed in figure 3 for the isotropic (A = 0) case and anisotropic (A = 25 and A = 50) cases.

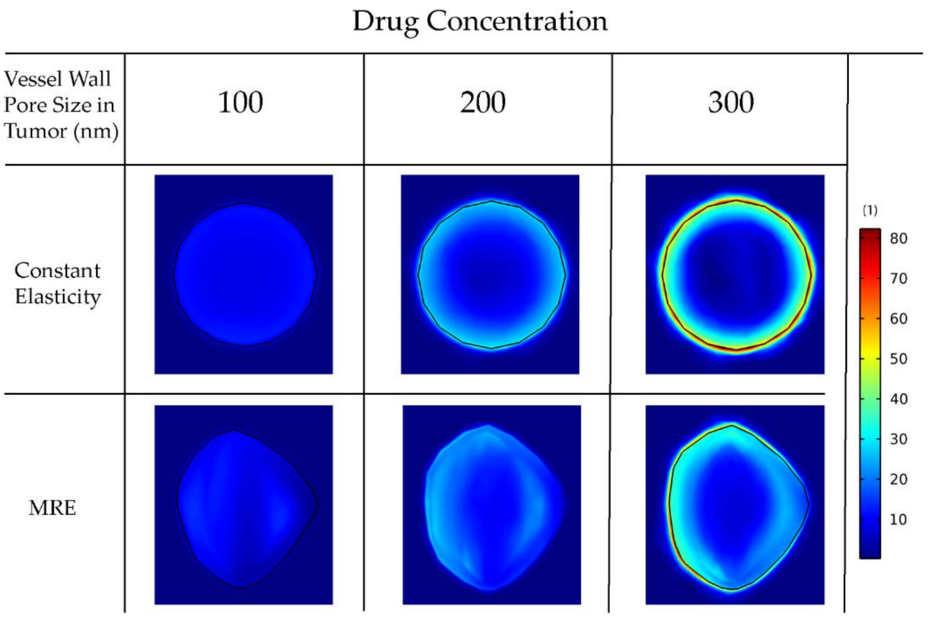

Figure S3. Second version of Figure 7 where the same colorbar was used for all figures.

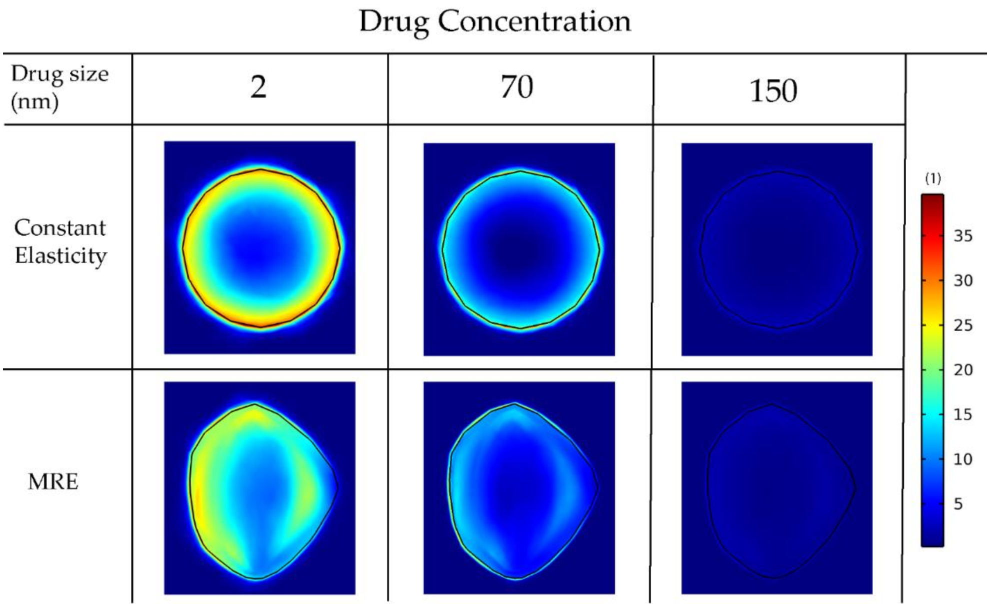

Figure S4. Second version of Figure 8 where the same colorbar was used for all figures.

References

1. Netti, P.A.; Berk, D.A.; Swartz, M.A.; Grodzinsky, A.J.; Jain, R.K. Role of Extracellular Matrix Assembly in Interstitial Transport in Solid Tumors. *Cancer Res.* **2000**, *60*, 2497–2503.
2. Kim, Y.; Stolarska, M.A.; Othmer, H.G. The Role of the Microenvironment in Tumor Growth and Invasion. *Prog. Biophys. Mol. Biol.* **2011**, *106*, 353–379, doi:10.1016/j.pbiomolbio.2011.06.006.
3. Hermann, P.C.; Huber, S.L.; Herrler, T.; Aicher, A.; Ellwart, J.W.; Guba, M.; Bruns, C.J.; Heeschen, C. Distinct Populations of Cancer Stem Cells Determine Tumor Growth and Metastatic Activity in Human Pancreatic Cancer. *Cell Stem Cell* **2007**, *1*, 313–323, doi:10.1016/j.stem.2007.06.002.
4. Casciari, J.J.; Sotirchos, S. V.; Sutherland, R.M. Variations in Tumor Cell Growth Rates and Metabolism with Oxygen Concentration, Glucose Concentration, and Extracellular PH. *J. Cell. Physiol.* **1992**, *151*, 386–394, doi:10.1002/jcp.1041510220.
5. Angeli, S.; Emblem, K.E.; Due-Tonnessen, P.; Stylianopoulos, T. Towards Patient-Specific Modeling of Brain Tumor Growth and Formation of Secondary Nodes Guided by DTI-MRI. *NeuroImage Clin.* **2018**, *20*, 664–673, doi:10.1016/j.nicl.2018.08.032.
6. Stylianopoulos, T.; Martin, J.D.; Snuderl, M.; Mpekris, F.; Jain, S.R.; Jain, R.K. Coevolution of Solid Stress and Interstitial Fluid Pressure in Tumors during Progression: Implications for Vascular Collapse. *Cancer Res.* **2013**, *73*, 3833–3841, doi:10.1158/0008-5472.CAN-12-4521.
7. Swanson, K.R.; Rostomily, R.C.; Alvord, E.C. A Mathematical Modelling Tool for Predicting Survival of Individual Patients Following Resection of Glioblastoma: A Proof of Principle. *Br. J. Cancer* **2008**, *98*, 113–119, doi:10.1038/sj.bjc.6604125.
8. Baxter, L.T.; Jain, R.K. Transport of Fluid and Macromolecules in Tumors. I. Role of Interstitial Pressure and Convection. *Microvasc. Res.* **1989**, *37*, 77–104, doi:10.1016/0026-2862(89)90074-5.
9. Schmidt, M.M.; Wittrup, K.D. A Modeling Analysis of the Effects of Molecular Size and Binding Affinity on Tumor Targeting. *Mol. Cancer Ther.* **2009**, *8*, 2861–2871, doi:10.1158/1535-7163.MCT-09-0195.
10. Mok, W.; Stylianopoulos, T.; Boucher, Y.; Jain, R.K. Mathematical Modeling of Herpes Simplex Virus Distribution in Solid Tumors: Implications for Cancer Gene Therapy. *Clin. Cancer Res.* **2009**, *15*, 2352–2360, doi:10.1158/1078-0432.CCR-08-2082.
11. Stylianopoulos, T.; Economides, E.-A.; Baish, J.W.; Fukumura, D.; Jain, R.K. Towards Optimal Design of Cancer Nanomedicines: Multi-Stage Nanoparticles for the Treatment of Solid Tumors. *Ann. Biomed. Eng.* **2015**, *43*, 2291–2300, doi:10.1007/S10439-015-1276-9.
12. Mpekris, F.; Angeli, S.; Pirentis, A.P.; Stylianopoulos, T. Stress-Mediated Progression of Solid Tumors: Effect of Mechanical Stress on Tissue Oxygenation, Cancer Cell Proliferation, and Drug Delivery. *Biomech. Model. Mechanobiol.* **2015**, *14*, 1391–1402, doi:10.1007/s10237-015-0682-0.
13. Pluen, A.; Boucher, Y.; Ramanujan, S.; McKee, T.D.; Gohongi, T.; Di Tomaso, E.; Brown, E.B.; Izumi, Y.; Campbell, R.B.; Berk, D.A.; et al. Role of Tumor-Host Interactions in Interstitial Diffusion of Macromolecules: Cranial vs. Subcutaneous Tumors. *Proc. Natl. Acad. Sci. U. S. A.* **2001**, *98*, 4628–4633, doi:10.1073/pnas.081626898.
14. Chauhan, V.P.; Stylianopoulos, T.; Martin, J.D.; PopoviÄ, Z.; Chen, O.; Kamoun, W.S.; Bawendi, M.G.; Fukumura, D.; Jain, R.K. Normalization of Tumour Blood Vessels Improves the Delivery of Nanomedicines in a Size-Dependent Manner. *Nat. Nanotechnol.* **2012**, *7*, 383–388, doi:10.1038/nnano.2012.45.
15. Késmárky, G.; Kenyeres, P.; Rábai, M.; Tóth, K. Plasma Viscosity: A Forgotten Variable. *Clin. Hemorheol. Microcirc.* **2008**, *39*, 243–246, doi:10.3233/CH-2008-1088.

16. Hobbs, S.K.; Monsky, W.L.; Yuan, F.; Roberts, W.G.; Griffith, L.; Torchilin, V.P.; Jain, R.K. Regulation of Transport Pathways in Tumor Vessels: Role of Tumor Type and Microenvironment. *Proc. Natl. Acad. Sci. U. S. A.* **1998**, *95*, 4607–4612, doi:10.1073/pnas.95.8.4607.
